# Psychotherapeutische Versorgung in Österreich: Kassenfinanzierte Psychotherapie für Menschen mit chronisch psychischen Erkrankungen im Jahresvergleich 2017 bis 2020

**DOI:** 10.1007/s00729-022-00194-9

**Published:** 2022-04-13

**Authors:** Friedrich Riffer, Magdalena Knopp, Claudia Oppenauer, Manuel Sprung

**Affiliations:** 1 Psychosomatisches Zentrum Waldviertel, Universitätsklinikum für Psychosomatische Medizin Eggenburg, Grafenberger Straße 2, 3730 Eggenburg, Österreich; 2 Psychosomatisches Zentrum Waldviertel, Rehabilitationsklinik Gars am Kamp, Kremserstraße 656, 3571 Gars am Kamp, Österreich; 3 Department für Psychologie und Psychodynamik, Fachbereich für Klinische Psychologie, Karl Landsteiner Privatuniversität für Gesundheitswissenschaften, Dr. Karl-Dorrek-Straße 30, 3500 Krems, Österreich

**Keywords:** Psychotherapie, Versorgung, Psychische Erkrankungen, Kassenplätze, Psychotherapy, Mental health care, Mental disorders, Public insurance funded psychotherapy

## Abstract

In Österreich wird die psychotherapeutische Versorgung im niedergelassenen Bereich finanziell über die Kostenzuschussregelung und kassenfinanzierten Psychotherapiestunden geregelt. Die vorliegende Studie untersucht, inwiefern sich der Anteil an selbstfinanzierten und kassenfinanzierten Psychotherapieeinheiten über die Jahre 2017–2020 unter Berücksichtigung des sozioökonomischen/krankheitsbezogenen und behandlungsbezogenen Status verändert hat. Hierfür wurde eine Stichprobe von 6387 Patient*innen mit psychischen Störungen im Rahmen einer stationären Behandlung befragt.

Der größte Teil (70 %) der Patient*innen ist seit mehr als zwei Jahren an einer psychischen Störung erkrankt und hatte bereits einen stationären Aufenthalt (46 %) oder ambulante psychotherapeutische Behandlung (82 %) in Anspruch genommen. Im Zuge der ambulanten psychotherapeutischen Vorbehandlung haben 45 % der Patient*innen einen Kassenplatz für Psychotherapie erhalten. Von den Patient*innen, die die Psychotherapie privat finanziert haben, hat der Großteil der Patient*innen (72 %) für eine psychotherapeutische Behandlung bis zu 100 € bezahlt – bei einem Anteil von 70 % von Patient*innen, die vor der stationären Behandlung arbeitsunfähig waren und 39 % von Patient*innen, deren monatliches Einkommen weniger als 1000 € ausmacht. Die Studiendaten zeigen auch, dass sich der Anteil der Patient*innen, die eine kassenfinanzierte Psychotherapie in Anspruch nehmen konnten seit 2017 nicht erhöht hat.

Die vorliegende Studie verdeutlicht, dass die Inanspruchnahme von ambulanter Psychotherapie und Kassenplätzen für Psychotherapie bei Patient*innen mit chronisch psychischen Erkrankungen in den letzten vier Jahren, trotz Erhöhung des Kassenzuschuss im Jahr 2018 und etwaiger Aufstockungen an Kassenplätzen, unverändert geblieben ist. Angesichts der limitierten Kassenplätze ist deshalb ein transparentes System für die Zuteilung von Kassenplätzen zu fordern, um die Inanspruchnahme von ambulanter Psychotherapie bei allen Patient*innen mit chronischen psychischen Erkrankungen zu ermöglichen.

## Abstract (en)

Outpatient psychotherapeutic care (private practice) in Austria is regulated financially through the cost subsidy regulation and health insurance funded psychotherapy hours. The present study examines the proportion of self-financed and health insurance funded psychotherapy units between the years 2017–2020, considering the socio-economic-/disease- and treatment related status of patients.

A sample of 6387 inpatients with a mental illness was surveyed during inpatient treatment.

The majority (70%) of inpatients had suffered from a mental illness for more than two years and had previously received inpatient (46%) or outpatient (82%) psychotherapeutic treatment. Of the patients with prior outpatient psychotherapeutic treatment, 45% received a health insurance funded psychotherapy. Of the patients who self-financed psychotherapy, the majority of patients (72%) paid up to € 100 for psychotherapeutic treatment—with 70% of patients who were unable to work prior inpatient treatment and 39% of patients whose monthly income is less than € 1000. The study data also show that the number of patients who have been able to use health insurance funded psychotherapy has not increased since 2017.

The present study illustrates that the use of outpatient psychotherapy and health insurance funded psychotherapy amongst patients with chronic mental illnesses remained unchanged in the last four years, despite the increase in the health insurance subsidy in 2018 and any increases in health insurance funded psychotherapy hours. Given the limited health insurance funded psychotherapy hours, a transparent system for the allocation of health insurance funded psychotherapy hours is required to enable the use of outpatient psychotherapy for all patients with chronic mental illnesses.

## Einleitung

Psychische Erkrankungen tragen im hohen Maße zur Krankheitslast bei und stellen für das österreichische Gesundheitssystem eine Herausforderung dar. Etwa jeder fünfte Mensch hat in den letzten 12 Monaten an einer psychischen Erkrankung gelitten, und ein Drittel der Allgemeinbevölkerung ist im Laufe des Lebens von psychischen Störungen betroffen (Steel et al. 2014). Die geschätzte 12-Monats-Prävalenz von psychischen Erkrankungen liegt europaweit bei 38,2 % (Wittchen et al. 2011). Aus einer WHO-Studie geht hervor, dass psychische Störungen zu den zehn Krankheiten zählen, die am häufigsten mit massiven Einschränkungen im Alltag und dem Verlust der Arbeitsfähigkeit einhergehen (Kessler et al. 2009). Die Folgen von psychischen Erkrankungen ziehen sowohl individuelle als auch volkswirtschaftliche Belastungen nach sich. Besonders indirekte Kosten von unbehandelten psychischen Erkrankungen durch Arbeitslosigkeit, verminderte Erwerbsfähigkeit, erhöhte Anzahl an Krankenstandstagen, sowie Frühpensionierungen belasten das Gesundheitssystem und die Volkswirtschaft. So lag etwa im Jahr 2020 die durchschnittliche Dauer der Krankenstände aufgrund psychischer Erkrankungen mit 42,1 Tagen deutlich über dem Durchschnittswert von 11,7 Tagen für alle Krankheitsarten insgesamt (Statistik Austria 2020). Auch jede dritte Frühpensionierung erfolgt aufgrund einer psychischen Erkrankung (Hauptverband der österreichischen Sozialversicherungsträger 2011). Der volkswirtschaftliche Gesamtschaden, den psychische Erkrankungen verursachen, beträgt laut der International Labour Organization etwa 3–4 % des Bruttonationalprodukts der EU-Mitgliedsstaaten (Gabriel und Liimatainen 2000). Auch für Österreich werden die durch psychische Erkrankungen verursachten direkten und indirekten Kosten auf 3,5 % des Bruttoinlandsprodukt, also ca. 13,1 Mrd. €, geschätzt (OECD 2015).

Im Hinblick auf die direkten Kosten für die psychotherapeutische Versorgung in Österreich sind aktuell nur wenige valide Daten verfügbar. Eine im Auftrag des Bundesministeriums für Soziales, Gesundheit, Pflege und Konsumentenschutz durchgeführte Erhebung zeigt, dass im Jahr 2014 die Krankenkassen 76,4 Mio. € für Psychotherapie aufgewendet hatten. Im Vergleich dazu wurden 2014 von den Krankenkassen 256 Mio. € für Psychopharmaka ausgegeben (Grabenhofer-Eggerth und Sator 2019). Demnach werden die Mittel der österreichischen Gesundheitskassen überwiegend für Psychopharmakotherapie ausgeben, obwohl die nachhaltige Wirksamkeit von Psychotherapie in vielen Studien bestätigt wurde (Cuijpers et al. 2020), und gesundheitsökonomische Studien auch ein positives Kosten-Nutzen-Verhältnis nach Abzug der Kosten für Psychotherapie aufzeigen (Margraf 2009).

In den vergangenen Jahren ist in Österreich auch eine Zunahme der Inanspruchnahme von Gesundheitsleistungen infolge psychischer Erkrankungen zu verzeichnen (Sagerschnig et al. 2018). Eine psychotherapeutische Behandlung kann auch zu Einsparungen von indirekten Kosten beitragen. Die Kosten für psychotherapeutische Behandlung werden durch die Einsparungen, aufgrund der Reduktion von Hospitalisierungen, Medikamenten und Produktivitätsverlusten, mehr als aufgewogen (Margraf 2009). Ein früher Diagnosezeitpunkt und eine rechtzeitige Behandlung sind weiters für die Eindämmung der Kosten entscheidend, wie z. B. Schneider und Dreer in einer Analyse der volkswirtschaftlichen Kosten von Burnout gezeigt haben (Schneider und Dreer 2013).

Gemäß dem Allgemeinen Sozialversicherungsgesetz der Republik Österreich ist eine Psychotherapie bei Vorliegen einer psychischen Erkrankung (nach der Internationalen statistischen Klassifikation der Krankheiten ICD-10) eine Pflichtleistung der österreichischen Gesundheitskasse(n). Es ist jedoch festzustellen, dass in Österreich nur ein Teil der Personen mit psychischen Erkrankungen eine psychotherapeutische Behandlung erhält (Riffer et al. 2021). Unzureichende finanzielle Mittel werden von den Betroffenen als häufige Gründe für eine ausbleibende Behandlung angegeben (Riffer et al. 2021). Die Ergebnisse einer aktuellen bevölkerungsrepräsentativen Umfrage zeigen, dass in Österreich nur 27 % der Personen mit psychischen Erkrankungen einen Kassenplatz für Psychotherapie erhalten. Weitere 21 % bezahlen die Psychotherapie gänzlich aus privaten Mitteln. Die Mehrheit der Betroffenen (52 %) muss die Kosten für ambulante Psychotherapie, bis auf einen Kostenzuschuss von 28 €, selbst tragen (Tanios et al. 2020). Der Kostenzuschuss für Psychotherapie wurde 2018 erstmal seit 27 Jahren von 21,80 € auf 28 € erhöht (Österreichische Gesundheitskasse 2020).

Selbst bei Patient*innen mit einer chronischen psychischen Erkrankung und Niedrigsteinkommen aufgrund von Arbeitslosigkeit oder Erwerbsunfähigkeit infolge ihrer psychischen Erkrankung erhält nur etwas mehr als die Hälfte (59 %) einen Kassenplatz für Psychotherapie (Riffer et al. 2018b). Bei durchschnittlichen Behandlungskosten von 80 bis 120 € pro psychotherapeutischer Behandlungseinheit können sich besonders einkommensschwache Menschen eine psychotherapeutische Behandlung sehr schwer leisten.

Die vorliegende Studie untersucht mögliche Veränderungen in der psychotherapeutischen Versorgungslage in Österreich und erforscht inwiefern sich das Verhältnis von selbstfinanzierten und kassenfinanzierten Psychotherapieeinheiten unter Berücksichtigung des sozioökonomischen/krankheitsbezogenen und behandlungsbezogenen Status verändert hat. Die Daten wurden über die Jahre 2017–2020 erhoben, um auch mögliche Veränderungen in der Versorgungslage über die Jahre hinweg, insbesondere auch in Bezug auf die Anhebung des Kostenzuschusses im Jahr 2018, untersuchen zu können.

## Methode

### Design

Im Rahmen einer naturalistischen Studie wurden Routinedaten, die im Psychosomatischen Zentrum Waldviertel (Universitätsklinikum Eggenburg und Rehaklinik Gars am Kamp) zwischen Juli 2017 und April 2020 gesammelt wurden, retrospektiv ausgewertet.

### Stichprobe

Die Stichprobe umfasst 6387 stationäre Patient*innen mit chronisch psychischen Erkrankungen, die im Zuge einer stationären psychotherapeutischen Behandlung zu ambulanter Psychotherapie befragt wurden. Das durchschnittliche Alter beträgt 47,9 Jahre, mit einem Frauenanteil von 65 %. Im Universitätsklinikum Eggenburg werden Patient*innen mit folgenden psychischen Störungen behandelt: ca. ein Drittel mit neurotischen, Belastungs- und somatoformen Störungen, ca. 20 % mit affektiven Störungen, ca. 10 % mit Persönlichkeits- und Verhaltensstörungen, ca. 10 % mit psychischen- und Verhaltensstörungen durch Alkohol und ca. 15 % mit Essstörungen (Burghardt et al. 2021; Riffer et al. 2017, 2018a). In der Rehaklinik in Gars am Kamp werden vorwiegend Patient*innen mit affektiven Störungen und neurotischen, Belastungs- und somatoformen Störungen behandelt.

### Behandlung

Das Universitätsklinikum Eggenburg ist ein multidisziplinäres und stationäres Behandlungszentrum für Menschen mit chronisch psychischen Erkrankungen. Die Klinik verfügt über 100 stationäre Betten und die interdisziplinäre Behandlung erfolgt in Kompetenzbereichen, die nach unterschiedlichen Störungsbildern gegliedert sind. Der stationäre Aufenthalt dauert im Durchschnitt zwischen 8 bis 12 Wochen, in denendie Patient*innen eine störungsspezifische und interdisziplinäre Therapie erhalten. In der psychiatrischen Rehabilitationsklinik Gars am Kamp beträgt die Aufenthaltsdauer standardmäßig 6 Wochen. Die Rehabilitationsklinik verfügt ebenso über 100 Betten und die interdisziplinäre Behandlung erfolgt teilweise störungsspezifisch.

### Erhebungsverfahren

Im Rahmen des routinemäßigen Outcome Monitorings (ROM (Egeter et al. 2018)) wurden zu Beginn und gegen Ende des stationären Aufenthaltes soziodemografische, sozioökonomische Informationen, gesundheits- und krankheitsbezogene Daten, sowie Informationen zur psychotherapeutischen Vorbehandlung und geplanter weiterführender Behandlung, erhoben. Das ROM erfolgt über das Health Evaluation System (CHES, Holzner et al. 2012). Symptombezogene Outcomes wurden mittels standardisierter und validierter Testverfahren, wie dem PHQ‑9 (Depressionssymptome, Kroenke et al. 2010), GAD‑7 (Angstsymptome, Spitzer et al. 2006), WHODAS (Funktionsfähigkeit, Küçükdeveci et al. 2013) und PHQ-15 (Somatische Symptome, van Ravesteijn et al. 2009) ermittelt.

### Statistische Datenauswertung

Alle Daten für die vorliegende Studie wurden mit dem CHES System erfasst und anschließend in pseudonymisierter Form mit dem Statistiksoftwareprogramm SPSS (Version 27.0) analysiert. Es wurden deskriptive Statistiken mit Häufigkeitsverteilungen und Mittelwerten für die verschiedenen Variablen berechnet. Die deskriptiven Statistiken wurden anschließend inferenzstatistisch mit *χ*^*2*^-Test, *t*-Tests und Kruskal-Wallis *H*-Test auf Signifikanz geprüft, mit einem Signifikanz-Niveau von *p* = 0,05.

## Ergebnisse

### Sozioökonomische und krankheitsbezogene Daten

Die Ergebnisse der sozioökonomischen und krankheitsbezogenen Angaben der Patient*innen sind in Tab. 1 dargestellt. Zum Zeitpunkt der Aufnahme waren 70,2 % der Patient*innen arbeitsunfähig und 11,9 % der Patient*innen waren in Frühpension. Mehr als ein Drittel (38,8 %) gab an, dass ihnen weniger als 1000 € im Monat zu Verfügung stehe. Es gaben 45,9 % der Patient*innen an in den letzten 12 Monaten bis zu zwei Wochen stationär im Krankenhaus gewesen zu sein und 19,9 % gaben an mehr als 20-Mal in den letzten sechs Monaten einen Arzt konsultiert zu haben. Die Patient*innen litten zum größten Teil seit mehr als zwei Jahren an einer psychischen Erkrankung (70,1 %).

**Table 1:** 

Eigenschaften	*n*	%
*Geschlecht*		
Weiblich	4157	65,1
Männlich	2230	34,9
*Alter in Jahren* (M = 47,09, SD = 11,23)		
25–44	1669	32,5
44–60	3135	61,1
60–75	313	6,1
75–90	12	0,2
*Erwerbsunfähigkeit*		
Ja	3598	70,2
Nein	1531	29,8
*Beziehungsstatus*		
Ledig/allein lebend	1604	30,1
Verheiratet	1815	34,0
In fester Partnerschaft	866	16,2
Getrennt/Geschieden	943	17,7
Verwitwet	109	2,0
*Eigenes Einkommen*		
Weniger als 1000 €	1067	38,8
1000–2000 €	1267	46,1
Mehr als 2000 €	416	15,1
*Höchste abgeschlossene Ausbildung*		
Kein Schulabschluss	92	1,7
Allgemeinbildende Pflichtschule	926	17,4
Lehre	1705	32,0
Berufsbildende mittlere Schule	905	17,0
Höhere Schule	970	18,2
Akademie	141	2,6
Hochschule (Universität, FH)	591	11,1
*Häufigkeit der Arztbesuche*^*a*^		
0- bis 5‑mal	948	17,9
6- bis 10-mal	1743	32,9
11- bis 20-mal	1558	29,4
Mehr als 20-mal	1054	19,9
*Wochen im Krankenhaus (stationär)*^*b*^		
Bis 2 Wochen	1023	45,9
2 bis 6 Wochen	703	31,5
Mehr als 6 Wochen	505	22,6

^a^In den letzten 6 Monaten

^b^In den letzten 12 Monaten

### Behandlungsbezogene Daten

Die Ergebnisse zur psychotherapeutischen Vorbehandlung und geplanten weiterführenden Behandlung sind in Tab. 2 und 3 angegeben. Eine ambulante psychotherapeutische Vorbehandlung hatten 4283 Patient*innen (81,7 %), wobei diese bei 53,9 % länger als ein Jahr dauerte. Eine weiterführende ambulante Psychotherapie hatten 88,1 % der Patient*innen geplant.

**Table 2:** 

Behandlungsbezogene Daten	Kassenplatz % (*n*)	Kein Kassenplatz % (*n*)	Gesamte Stichprobe % (*N*)
*Stationäre Vorbehandlung*			
Ja	59,0 (1138)	42,6 (984)	46,1 (2424)
Nein	41,0 (791)	57,4 (1328)	53,9 (2839)
*Anzahl psychotherapeutischer Behandlungen (ambulant), in den letzten 12 Monaten*			
1- bis 5‑mal	28 (505)	26,7 (589)	27,3 (1100)
6- bis 10-mal	19,7 (355)	21,7 (477)	20,8 (838)
11- bis 20-mal	19,5 (352)	23,6 (520)	21,8 (877)
Mehr als 20-mal	32,7 (590)	28 (616)	30,2 (1217)
*Gesamtdauer psychotherapeutischer Vorbehandlung (ambulant)*			
Bis 3 Monate	22 (416)	22,5 (516)	22,2 (936)
Bis 1 Jahr	19,6 (371)	27,6 (635)	23,9 (1010)
Länger als 1 Jahr	58,4 (1107)	49,9 (1146)	53,9 (2273)
*Wartezeit auf Kassenplatz*			
Bis 1 Woche	19,6 (258)	–	19,6 (358)
Bis 5 Wochen (1 Mo.)	39,2 (715)	–	39,3 (716)
Bis 15 Wochen (3 Mo.)	22,1 (402)	–	22,0 (402)
Bis 30 Wochen (6 Mo.)	7,4 (135)	–	7,4 (135)
Mehr als 30 Wochen (6 Mo.)	11,7 (213)	–	11,7 (213)
*Kosten für nicht Kassen-finanzierte Psychotherapie (je Einheit/Stunde)*			
Bis € 50	–	15,3 (349)	15,3 (350)
Bis € 100	–	71,7 (1638)	71,7 (1639)
Mehr als € 100	–	13,0 (298)	13,0 (298)
*Eigenes Einkommen*			
Weniger als 1000 €	50,3 (502)	28,5 (336)	38,8 (1067)
1000–2000 €	42,4 (424)	49,3 (581)	46,1 (1267)
Mehr als 2000 €	7,3 (73)	22,2 (1178)	15,1 (416)
*Erwerbsunfähigkeit*			
Ja	71,8 (1328)	68,6 (1545)	70,2 (3598)
Nein	28,2 (522)	31,4 (706)	29,8 (1531)

**Table 3:** 

Geplante psychotherapeutische Weiterbehandlung	Kassenplatz % (*n*)	Kein Kassenplatz % (*n*)	Gesamte Stichprobe % (*N*)
Ja, stationär	5,7 (283)	2,5 (46)	4,0 (155)
Ja, ambulant	89,4 (1308)	91,3 (1663)	88,1 (3425)
Keine weiterführende Behandlung geplant	4,9 (72)	6,2 (113)	7,9 (308)
*Weiterbehandlungstermin bereits vereinbart*			
Ja	60,3 (838)	56,6 (967)	54,9 (1962)
Nein	39,7 (551)	43,4 (740)	45,1 (1612)

Von jenen Patienten*innen mit einer ambulanten psychotherapeutischen Vorbehandlung hatten 1932 (45,5 %) einen Kassenplatz und 2318 (54,5 %) keinen Kassenplatz. Vergleicht man Patient*innen mit/ohne einen Kassenplatz, hatten Patient*innen mit einem Kassenplatz ein signifikant geringeres Einkommen als jene ohne Kassenplatz (*t* = 12,69, *p* = 0,022). Zudem hatten Patient*innen mit einem Kassenplatz signifikant häufiger eine stationäre Vorbehandlung als jene ohne (*t* = −10,83, *p* = 0,041). Von jenen die keinen Kassenplatz hatten, zahlten 71,7 % bis zu 100 € pro Psychotherapieeinheit.

In Tab. 4 sind die privaten Kosten für nicht kassenfinanzierte Psychotherapie, sowie der Anteil der Patient*innen mit einer psychotherapeutischen Vorbehandlung vor und nach der Erhöhung des Kostenzuschusses am 01.09.2018 aufgelistet. Der Anteil der privaten Kosten für eine Psychotherapieeinheit ist demnach auch nach der Erhöhung des Kostenzuschusses 2018 unverändert hoch.

**Table 4:** 

Behandlungsbezogene Daten	Vor Erhöhung des Kostenzuschusses	Nach Erhöhung des Kostenzuschusses
*Kosten für nicht Kassen-finanzierte Psychotherapie (je Einheit/Stunde)*		
Bis € 50	15,6 (116)	15,2 (235)
Bis € 100	72,8 (542)	71,1 (1097)
Mehr als € 100	11,7 (87)	13,7 (211)
*Psychotherapeutische Vorbehandlung (ambulante)*		
Ja	82,3 (1399)	81,4 (2884)
Nein	17,7 (300)	18,6 (661)

**Figure 1:**
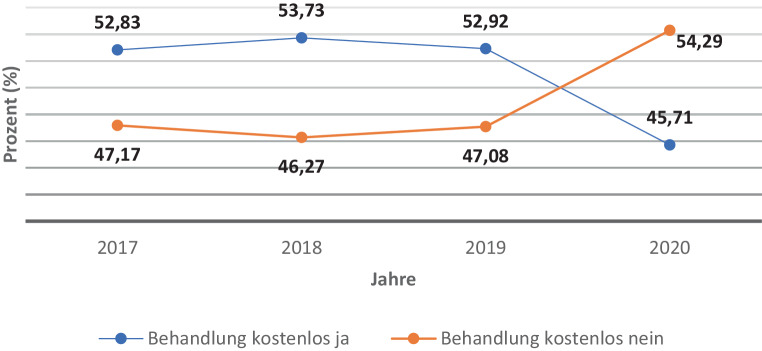

Der Anteil der Patient*innen mit bzw. ohne Kassenplatz ist in Abb. 1 im Jahresvergleich von 2017 bis 2020 dargestellt. Es zeigt sich demnach zwischen 2017 und 2020 keine signifikanten Veränderungen in der Inanspruchnahme von Kassenplätzen (Kruskal-Wallis *H* (3) = 1,681, *p* = 0,641).

Im Vergleich von Patient*innen mit versus ohne Kassenplatz zeigten sich keine signifikanten Unterschiede in Hinblick auf den Schweregrad der krankheitsbezogenen Symptomatik, z. B. depressiv/ängstliche Symptome (siehe Tab. 5).

**Table 5:** 

	Mit Kassenplatz		Ohne Kassenplatz		Signifikanz t‑Test	
Fragebogen	M	SD	M	SD	*t* (df)	*p*
PHQ‑9	14,61	5,83	13,46	5,73	−6,41 (4225)	0,154
PHQ-15	13,29	5,91	11,92	5,71	−7,63 (4221)	0,179
GAD‑7	11,74	4,95	10,91	4,99	−5,42 (4226)	0,789
WHODAS	20,01	9,05	17,69	8,73	−8,44 (4201)	0,485

*p ≤ 0,001

## Diskussion

Die Ergebnisse zeigen, dass Patient*innen mit chronischer psychischer Erkrankung, trotz teilweise wiederholter stationärer psychotherapeutischer Aufenthalte nur zur Hälfte (54,5 %), einen Kassenplatz für Psychotherapie in Anspruch nehmen können und vielfach mehrere Monate auf diesen warten müssen. Patient*innen mit und ohne Kassenplatz weisen eine vergleichbar hohe Symptombelastung auf. Der Schweregrad der Symptome steht demnach in keinem Zusammenhang mit der Inanspruchnahme eines Kassenplatzes. Angesichts der limitierten Kassenplätze ist deshalb ein transparentes System für die Zuteilung von Kassenplätzen wünschenswert, insbesondere auch unter Berücksichtigung der Chronifizierung und des Schweregrads der Symptome.

Knapp 30 % der Patient*innen ohne Kassenplatz haben ein geringes Einkommen (weniger als 1000 € im Monat). Die privat finanzierten Kosten für Psychotherapie (ca. 4000 € pro Jahr) stellen eine erhebliche finanzielle Belastung dar (Schosser et al. 2021). Die Studienergebnisse zeigen weiters, dass sich im Zusammenhang mit der Erhöhung des Kostenzuschusses der Anteil der Patient*innen mit chronisch psychischen Erkrankungen, die Psychotherapie in Anspruch nehmen, nicht verändert hat und die finanziellen Belastungen durch die privat finanzierten Kosten unverändert hoch sind.

Es zeigt sich auch, dass der Anteil der Patient*innen mit chronisch psychischen Erkrankungen, die einen Kassenplatz in Anspruch nehmen konnten, im Zeitraum zwischen 2017–2020 trotz Erhöhung des Kassenzuschuss und etwaiger Aufstockungen an Kassenplätzen unverändert geblieben ist. Die Prävalenz von psychischen Erkrankungen hat jedoch seit der COVID-19 Pandemie zugenommen (Duarte 2021) und die subjektive Belastung und der Behandlungsbedarf sind bei Patient*innen mit chronischen psychischen Erkrankungen ungleich höher als bei Patient*innen mit physischen Erkrankungen (Oppenauer et al. 2021).

Die Wirksamkeit von Psychotherapie ist gut belegt (Cuijpers et al. 2020). Es ist bedauerlich, dass in Österreich zwar fast alle Patient*innen mit chronischen psychischen Erkrankungen eine Psychopharmaka Therapie in Anspruch nehmen können, jedoch nur die Hälfte eine Psychotherapie in Anspruch nehmen kann (Grabenhofer-Eggerth und Sator 2019; Hauptverband der österreichischen Sozialversicherungsträger 2011). Die Effekte von Psychotherapie haben sich zudem im Vergleich zur Psychopharmaka Therapie als langfristiger erwiesen (Cuijpers et al. 2013). Es ist daher für die langfristige Genesung von Menschen mit psychischen Krankheiten notwendig neben Psychopharmakotherapie auch eine Psychotherapie zu ermöglichen.
